# Supplementary material for: Interface Probing by Dielectric Frequency Dispersion in Carbon Nanocomposites
Source: Sci Rep. 2018 Sep 28;8:14547. doi: 10.1038/s41598-018-32452-9 (PMC6162296; doi:10.1038/s41598-018-32452-9)
Supplement: Supplementary file 1 — Supplementary information [file 41598_2018_32452_MOESM1_ESM.pdf]

Supplementary Information

Interface Probing by Dielectric Frequency Dispersion in Carbon Nanocomposites

Yuhan Li, Faxiang Qin*, Diana Estevez, Huan Wang, Hua-Xin Peng.

Institute for Composites Science Innovation (InCSI), School of Material Science and Engineering, Zhejiang University, Zheda Road, Hangzhou, 310027, China

*E-mail: faxiangqin@zju.edu.cn

1. Backgrounds on dielectric spectroscopy and dielectric relaxation

The interaction between MWCNTs/SE nanocomposites and electromagnetic waves is mainly contributed by its dielectric properties, which can be expressed by complex permittivity (ε=ε'-$i\varepsilon$''), in which the real part ε' is associated with the electromagnetic energy storage and the ε'' is correlated to the energy dissipation within a material resulting from conduction, resonance, and relaxation mechanisms[1]. Polarization occurs as charges reorganize when applying electric field, contributing to the interaction among dipoles and the forming of overall dielectric properties. In the case of changing electric field, dielectric relaxation forms due to the movement of dipoles or electric charges, indicating the delay between polarization and electric field. Various polarization mechanism and relaxation process in material are often correlated with different length and time scales, making dielectric spectroscopy suitable for investigating nanocomposites dynamics[2].

The classical Debye model on dielectric relaxation is expressed as:

$$\varepsilon^*(\omega)=\varepsilon_\infty+\frac{\Delta\varepsilon}{1+i\omega\tau} \quad (1)$$

where ε_∞ is the permittivity at high frequency limit, $\Delta\varepsilon=\varepsilon_s-\varepsilon_\infty$ and ε_s is the static permittivity at low frequency and τ is the characteristic relaxation time[3], and empirical expression is developed based on Debye equations by Havriliak and Negami as shown in the main article.

The dielectric properties of nanocomposites are inextricable with the interfacial characteristics in terms of their influence on the permittivity, breakdown strength and relaxation dynamics[4-6]. The relaxation process concerning the existence of interface in heterogeneous materials is described by Maxwell-Wagner process, in which a representative example is the interface formed by the dispersion of conductive fillers in an insulating matrix. The dynamics of charge distribution within these systems will occur over a timescale determined by the different characteristics, leading to interesting dielectric properties[7]. In such systems, how quickly the relaxation process forms depends

on the electrostatic and electrophoretic forces, on the size of the dissociable or polar species, and on electrical forces[8], which has a significant connection with the interfacial chemistry and interfacial dynamics. Thereby, the dispersion and morphology of the nano-fillers may affect the interfacial dynamics[9]. In our research, the interfacial chemistry and the dispersion are the two main factors in determining the change in dielectric spectra in the CNTs/SE nanocomposites. The increased agglomerates surface area due to larger cluster size could slow down the dielectric process and result in larger relaxation time. Despite the complexity and difficulty in quantifying the interfacial chemical properties, research on interfacial correlated dielectric mechanisms are often based on experimental phenomena. Interfacial relaxation in composites is believed to relate with filler-matrix interaction and restricted polymer chains. Singha et.al have demonstrated the change of epoxy nature in TiO_2/epoxy nanocomposite by the alteration of permittivity at different frequency[10]. Zhenghai Tang et.al have observed the pattern change in the dielectric spectra of interfacial relaxation process, in which signal cannot be detected in some nanocomposites with poor interfacial adhesion[11]. Thereby, the variation in intensity and position of dielectric relaxation peak in dielectric spectra could be taken as an indication for structural changes in nanocomposites.

2. Dielectric frequency dispersion of carbon nanocomposites at representative low and high filler content

The complex dielectric spectra of CNTs/silicone elastomer nanocomposites with different filler contents (f=0.12 vol% and f=0.93 vol%) were shown in Figure S1. For nanocomposites with a filler content of 0.12 vol%, relaxation peak was not observed for MWCNTs/SE due to weak interface and filler-matrix interaction. With increasing CNTs contents(f=0.93 vol%), the increased conductivity and agglomeration could affect the dielectric response significantly, resulting in overshadowing of

interface-related relaxation peak in the dielectric spectra.

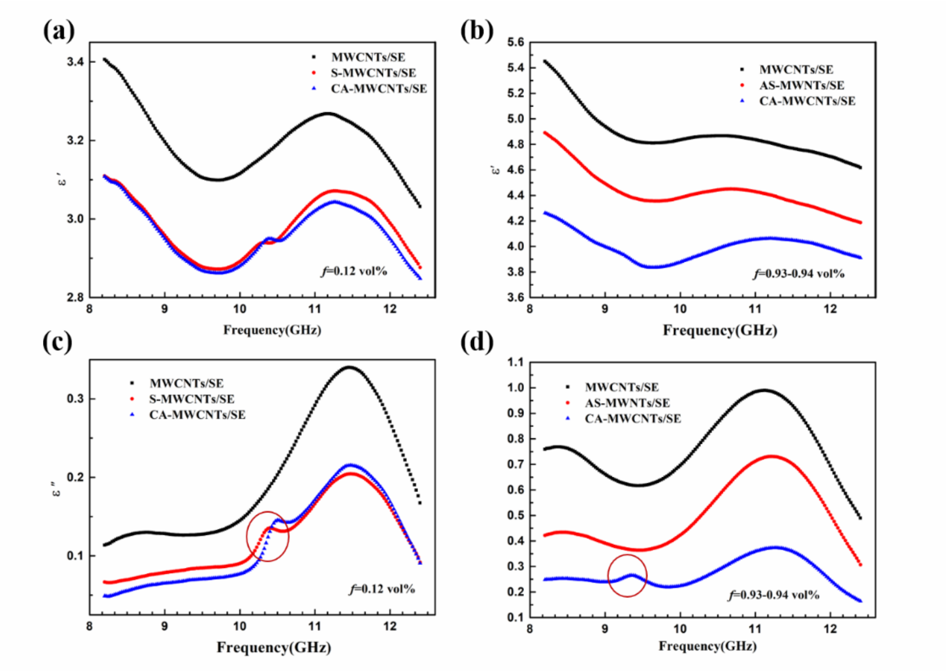

Figure S1. Frequency dependence of dielectric permittivity with different filler contents: (a) real part, *f*=0.12 vol%. (b) real part, *f*=0.93-0.94 vol%. (c) imaginary part, *f*=0.12 vol%. (d) imaginary part, *f*=0.93-0.94 vol%.

3. Statistical analysis on the dispersion state and agglomerates size distribution

Area ratio and agglomerates size distribution are used as a relative measurement of the agglomerated area[12,13]. The initial dispersion state for samples with different modification method is shown in Figure S2(a). A distinct difference was observed for the area ratio concerning samples with different modification methods (Figure S2(b)), in which CA-MWCNTs/SE shows an agglomerated ratio of only 2.3%, indicating the overall good dispersion state. Comparing with MWCNTs/SE (16.8%), AS-MWCNTs/SE is more spatially extensive (19.3%) due to the loose entanglement of CNTs fillers. Correspondingly, CNTs agglomerates in CA-MWCNTs/SE are mostly located in the size range of 0-5 μm. AS-MWCNTs/SE has larger ratio over MWCNTs /SE in the size range of 0-5 μm and 5-10 μm, while MWCNTs /SE has more aggregates above 10 μm than the other two samples, showing the lowest dispersion degree (Figure S2(c)).

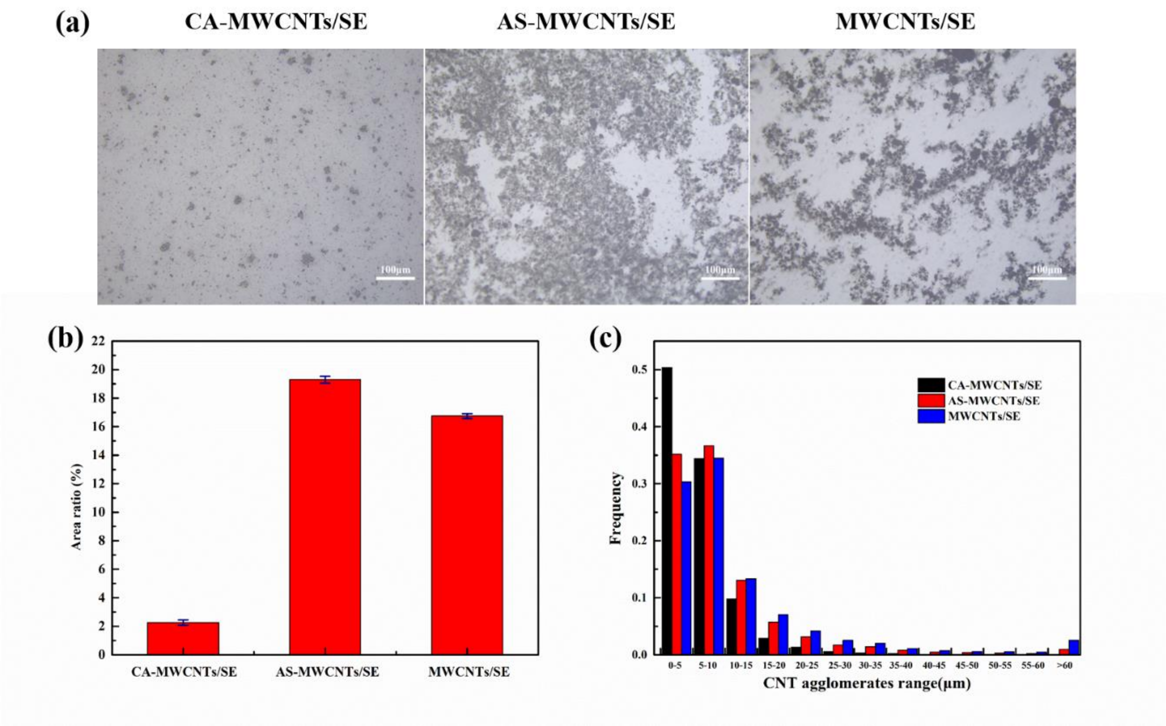

Figure S2. Statistical analysis on the dispersion state and agglomerates size distribution in CNTs/SE nanocomposites (f=0.46-0.50 vol%): (a) Optical microscopy images (200 ×) for nanocomposites with different interface modification methods. (b) Statistical agglomeration area ratio for each sample prepared by different methods. (c) Size distribution (each size class has a width of 5 μm) of MWCNTs agglomerates.

4. Dynamic mechanical analysis for characterizing filler-matrix interaction

DMA was used for analyzing interfacial interaction (Figure S3). Storage modulus was increased via modification by surfactant due to better dispersion. As the crosslinking of silicone elastomer was influenced by coupling agent, decrease in storage modulus was observed. Meanwhile, improved T_g was found for both modification methods.

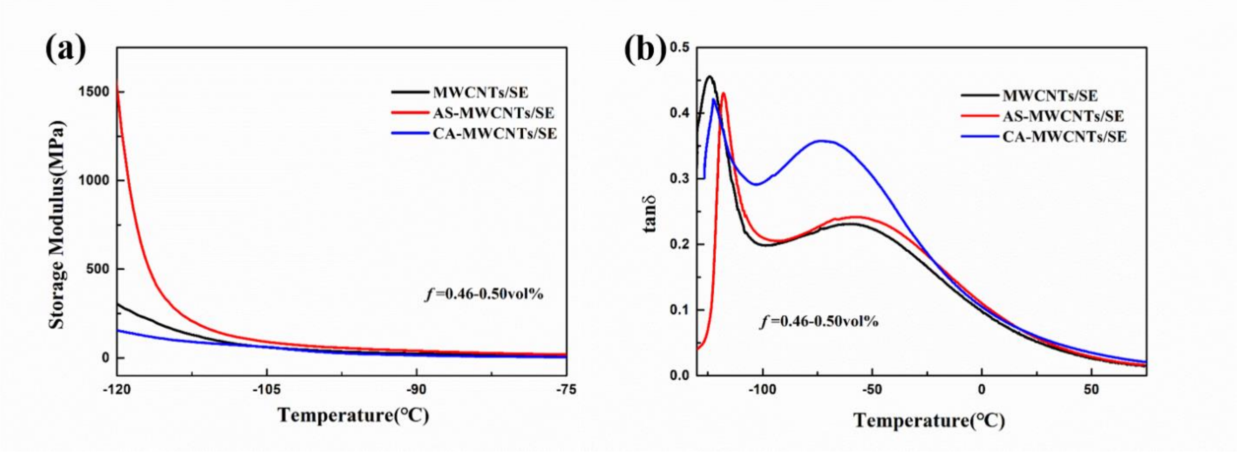

Figure S3. DMA curves of CNTs/silicone elastomer nanocomposites with pristine or modified CNTs: (a) Storage modulus of CNTs nanocomposites. (b) tan δ of CNTs nanocomposites

5. Raman spectra for characterizing interfacial interaction

Figure S4 shows the Raman shift of both raw MWCNTs and nanocomposites with different interfacial condition. Comparing to raw MWCNTs, the blueshift of D and G peaks is caused by coating of polymer on the surface of MWCNTs, which affects the vibration movements of original C-C bands. However, nanocomposites prepared with different modification methods do not show any indicative variation in peak position.

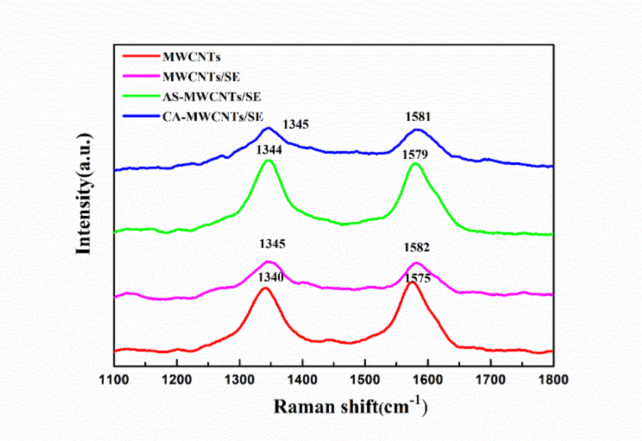

Figure S4. Raman shift showing the D peak and G peak of raw MWCNTs and MWCNTs/silicone elastomer nanocomposites (*f*=0.46%-0.50).

6. Evolution of DS with extended cyclic loading (to 80 cycles)

Cyclic loading cycles have been extended to 80 cycles for AS-MWCNTs/SE and CA-MWCNTs/SE to further investigate on the evolution of high frequency dielectric spectroscopy, as presented in Figure S5 and Figure S6 together with their cyclic tensile curves. For AS-MWCNTs/SE, the relaxation peak has disappeared at 10 cycles and reappeared after 20 times, and the appear-re-disappear pattern is reproduced from 60-80 loading cycles, which means that the reconstruction process still exists beyond 50 cycles of tensile loading. The relaxation peak around 10.5 GHz for CA-MWCNTs/SE is decreased upon increasing loading cycles till disappeared at 30 cycles, and remains unchanged eventually, which is consistent with the analyses on the irreversible

breakage of interfacial bonding. According to the tensile curves displayed in Figure S5 and Figure S6, stress-strain curves for different cycles overlaps with one another and no obvious changes upon increasing loading cycles is observed in our experiment, which can be the result of relatively weak interface in the investigated nanocomposites and limited sensitivity of tensile tests.

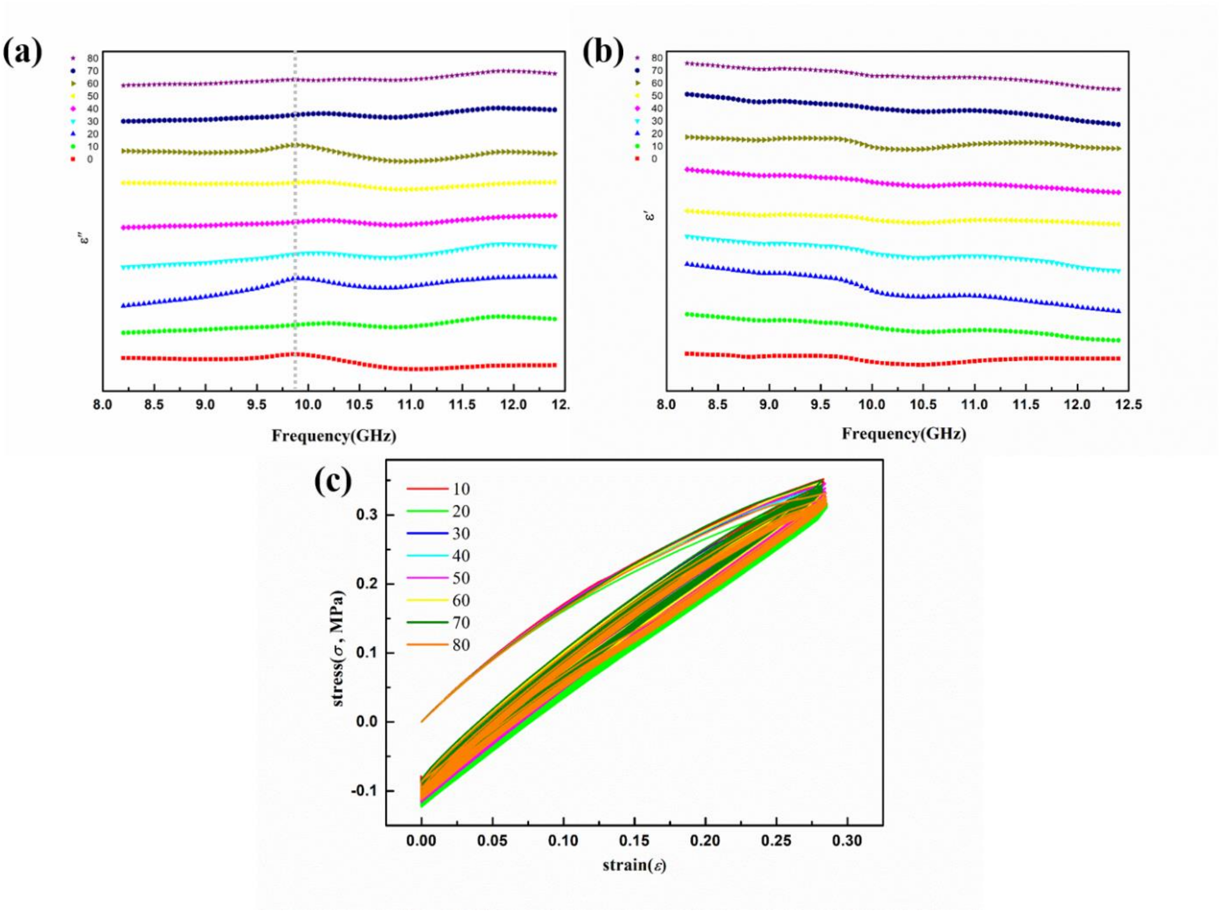

Figure S5. Variation of complex dielectric frequency spectra (8.2 GHz-12.4 GHz) for AS-MWCNTs/SE nanocomposites (*f*=0.47 vol%) with extended cyclic tensile loading (10, 20, 30, 40, 50, 60, 70, 80 cycles, strain averages 28% for each set of cycles) (a) imaginary part. (b) real part. (c) stress-strain curves for each set of cyclic tensile loading.

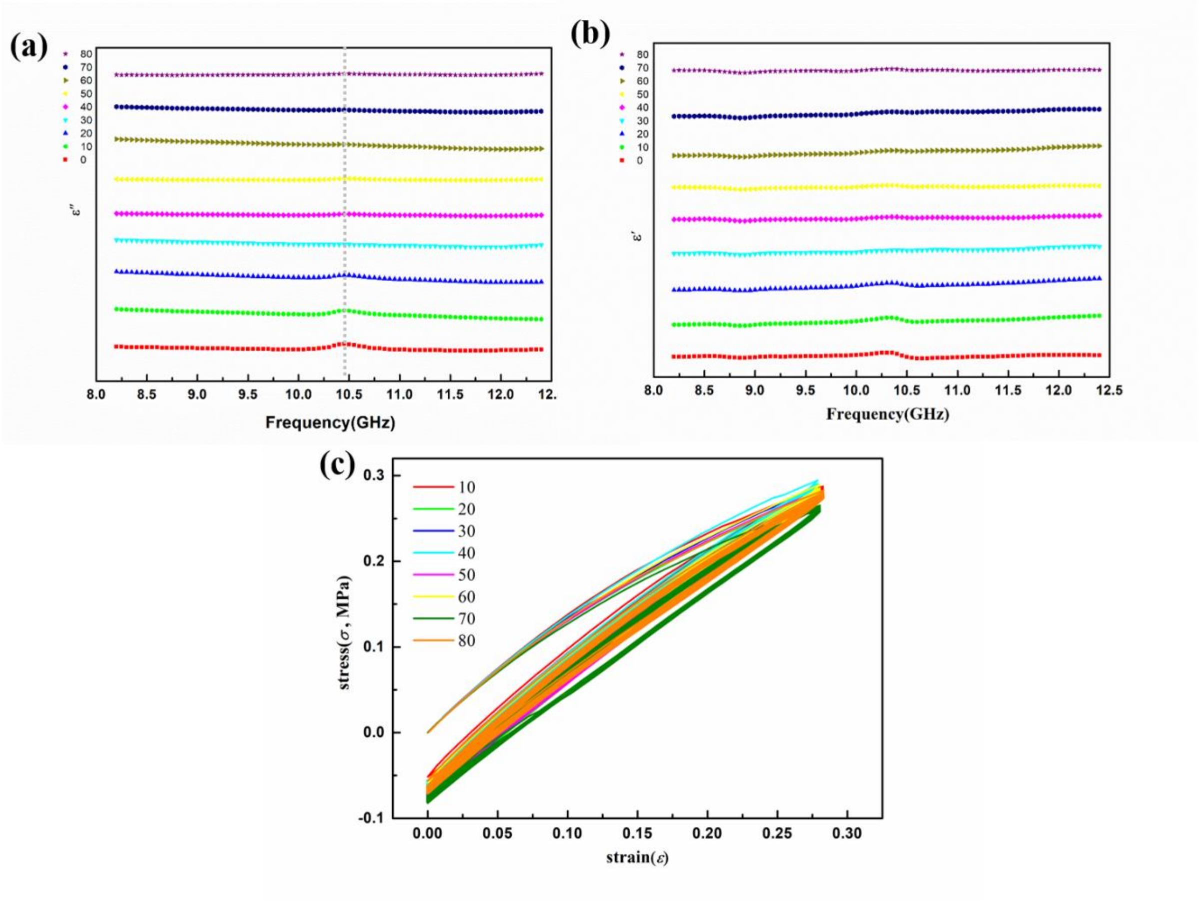

Figure S6. Variation of complex dielectric frequency spectra (8.2 GHz-12.4 GHz) for CA-MWCNTs/SE nanocomposites (*f*=0.47 vol%) with extended cyclic tensile loading (10, 20, 30, 40, 50, 60, 70, 80 cycles, strain averages 28% for each set of cycles) (a) imaginary part. (b) real part. (c) stress-strain curves for each set of cyclic tensile loading.

References

1. Achour, M.E., Brosseau, C. & Carmona, F. Dielectric relaxation in carbon black-epoxy composite materials. *J. Appl. Phys.* **103,** 094103(2008).
2. Mijović, J., Lee, H., Kenny, J. & Mays, J. Dynamics in polymer-silicate nanocomposites as studied by dielectric relaxation spectroscopy and dynamic mechanical spectroscopy. *Macromolecules* **39,** 2172-2182(2006).
3. Debye, P. *Polar Molecules* (Dover, 1945).
4. O'Konski, C. T. Electric properties of macromolecules. V. Theory of ionic polarization in polyelectrolytes. *J. Phys. Chem.* **64,** 605–619(1960).
5. Roy, M., Nelson, J. K., MacCrone, R. K., Schadler, L. S., Reed, C. W. & Keefe, R. Polymer nanocomposite dielectrics-the role of the interface. *IEEE Trans. Dielectr. Electr. Insul.* **12,** 629–643(2005).
6. Chew, W. C. & Sen, P. N. Dielectric Enhancement due to electrochemical double layer: thin double layer approximation. *J. Chem. Phys*. **77,** 4683–4693(1982).
7. Stuerga, D. & Delmotte, M. *Microwave in Organic Synthesis* 1-61 (Wiley-VCH, 2003).

8. Nelson, J. K. *et al. Dielectric Polymer Nanocomposites* (Springer, 2010).
9. Polizos, G., Tuncer, E., Tomer, V., Sauers, I., Randall, C.A. & Manias, E. *Nanoscale Spectroscopy with Applications* 93-130 (CRC, 2013).
10. Singha, S., Thomas, M.J. and Kulkarni, A. Complex permittivity characteristics of epoxy nanocomposites at low frequencies. *IEEE Trans. Dielectr. Electr. Insul.* **17,** 1249-1258(2010).
11. Tang, Z., Zhang, L., Feng, W., Guo, B., Liu, F. & Jia, D. Rational design of graphene surface chemistry for high-performance rubber/graphene composites. *Macromolecules* **47,** 8663-8673 (2014).
12. Kasaliwal, G.R., Pegel, S., Göldel, A., Pötschke, P. & Heinrich, G. Analysis of agglomerate dispersion mechanisms of multiwalled carbon nanotubes during melt mixing in polycarbonate. *Polymer* **51,** 2708-2720(2010).
13. Alig, I. *et al.* Establishment, morphology and properties of carbon nanotube networks in polymer melts. *Polymer* **53,** 4-28(2012).
